# Supplementary material for: Impact of old age on resectable colorectal cancer outcomes
Source: PeerJ. 2019 Feb 15;7:e6350. doi: 10.7717/peerj.6350 (PMC6378948; doi:10.7717/peerj.6350)
Supplement: Supplemental Information 3 [file peerj-07-6350-s003.docx]

| Risk Factors | Univariate analysis | | Multivariate analysis | |
| --- | --- | --- | --- | --- |
|  | HR (95%CI) | *P* ^b^ | HR (95%CI) | *P* ^b^ |
| Age |  |  |  |  |
| <70 | 1 |  | 1 |  |
| ≥70 | 1.48(1.43-1.53) | <0.001 | 1.66(1.6-1.72) | <0.001 |
| Gender |  |  |  |  |
| Female | 1 |  | 1 |  |
| Male | 1.09(1.05-1.12) | <0.001 | 1.16(1.12-1.20) | <0.001 |
| Marital status |  |  |  |  |
| Married | 1 |  | 1 |  |
| Unmarried | 1.45(1.39-1.52) | <0.001 | 1.33(1.27-1.40) | <0.001 |
| Divorced | 1.44(1.38-1.49) | <0.001 | 1.32(1.27-1.37) | <0.001 |
| Race |  |  |  |  |
| White | 1 |  | 1 |  |
| Black | 1.38(1.32-1.45) | <0.001 | 1.38(1.32-1.45) | <0.001 |
| Other | 0.94(0.88-0.99) | 0.027 | 0.90(0.85-0.95) | 0.426 |
| Location |  |  |  |  |
| Left | 1 |  | 1 | - |
| Right | 0.98(0.95-1.02) | 0.286 | 1.02(0.98-1.06) | <0.001 |
| Histology |  |  |  |  |
| [Adenocarcinoma](D:/360Downloads/Youdao/Dict/6.3.69.8341/resultui/frame/javascript:void(0);) | 1 |  | 1 |  |
| [Mucinous](D:/360Downloads/Youdao/Dict/6.3.69.8341/resultui/frame/javascript:void(0);) [adenocarcinoma](D:/360Downloads/Youdao/Dict/6.3.69.8341/resultui/frame/javascript:void(0);) | 1.22(1.16-1.28) | <0.001 | 1.13(1.07-1.18) | <0.001 |
| [Signet ring](D:/360Downloads/Youdao/Dict/6.3.69.8341/resultui/frame/javascript:void(0);) [cell](D:/360Downloads/Youdao/Dict/6.3.69.8341/resultui/frame/javascript:void(0);) [carcinoma](D:/360Downloads/Youdao/Dict/6.3.69.8341/resultui/frame/javascript:void(0);) | 3.45(3.09-3.85) | <0.001 | 1.71(1.53-1.91) | <0.001 |
| Differentiated grade |  |  |  |  |
| Grade I | 1 |  | 1 |  |
| Grade II | 1.37(1.28-1.48) | <0.001 | 1.23(1.15-1.33) | <0.001 |
| Grade III | 2.49(2.31-2.69) | <0.001 | 1.63(1.51-1.77) | <0.001 |
| T-classification ^a^ |  |  |  |  |
| T1 | 1 |  | 1 |  |
| T2 | 0.77(0.70-0.85) | <0.001 | 0.96(0.87-1.06) | 0.407 |
| T3 | 2.16(1.99-2.34) | <0.001 | 2.18(2.00-2.37) |  |
| T4 | 5.44(5.00-5.92) | <0.001 | 4.82(4.40-5.27) | <0.001 |
| N- classification ^a^ |  |  |  |  |
| N0 | 1 |  | 1 |  |
| N1 | 2.10(2.02-2.18) | <0.001 | 2.10(2.01-2.19) | <0.001 |
| N2 | 4.07(3.91-4.24) | <0.001 | 4.07(3.89-4.27) | <0.001 |
| nLN |  |  |  |  |
| 0 | 1 |  | 1 |  |
| 0-2 | 0.53(0.47-0.60) | <0.001 | 0.45(0.40-0.52) | <0.001 |
| 3-5 | 0.58(0.54-0.63) | <0.001 | 0.43(0.39-0.47) | <0.001 |
| 6-11 | 0.54(0.51-0.58) | <0.001 | 0.32(0.30-0.35) | <0.001 |
| ≥12 | 0.44(0.42-0.47) | <0.001 | 0.23(0.21-0.24) | <0.001 |
| CT |  |  |  |  |
| Yes | 1 |  | 1 |  |
| No | 1.54(1.49-1.59) | <0.001 | 0.81(0.78-0.85) | <0.001 |
| RT |  |  |  |  |
| Yes | 1 |  | 1 |  |
| No | 1.24(1.19-1.29) | <0.001 | 1.20(1.15-1.26) | <0.001 |

^a^ T classification according to 7^th^ AJCC staging system.

^b^ *P* values obtained from the χ2 test. All statistical tests were two-sided.

Abbreviations: PSM: propensity score matching; nLN: number of lymph nodes; CT: chemotherapy treatment; RT: radiotherapy treatment; HR: hazard ratio.

Left includes rectum, rectosigmoid junction, sigmoid colon, descending colon and splenic flexure.

Right includes transverse colon, hepatic flexure, ascending colon, cecum, and appendix.
