# Supplementary material for: Impact of old age on resectable colorectal cancer outcomes
Source: PeerJ. 2019 Feb 15;7:e6350. doi: 10.7717/peerj.6350 (PMC6378948; doi:10.7717/peerj.6350)
Supplement: Supplemental Information 4 [file peerj-07-6350-s004.docx]

| Characteristics | Younger(<70yrs)  Before PSM | Older(≥70yrs)  Before PSM | SD | Younger(≤70yrs)  After PSM | Older(≥70yrs)  After PSM | SD |
| --- | --- | --- | --- | --- | --- | --- |
|  | *N* (%) | *N* (%) |  | *N* (%) | *N* (%) |  |
| Gender |  |  |  |  |  |  |
| Female | 17301(53.65) | 14945(46.35) | 0.15 | 11983(49.02) | 12460(50.98) | 0.04 |
| Male | 21175(61.02) | 13525(38.98) | 0.45 | 12997(50.93) | 12520(49.07) | 0.04 |
| Marital status |  |  |  |  |  |  |
| Married | 26514(62.08) | 16192(37.92) | 0.50 | 16465(51.51) | 15497(48.49) | 0.06 |
| Single | 5019(69.19) | 2235(30.81) | 0.83 | 2317(50.90) | 2235(49.10) | 0.04 |
| Divorced | 6943(40.87) | 10043(59.13) | 0.37 | 6198(46.10) | 7248(53.90) | 0.16 |
| Race |  |  |  |  |  |  |
| White | 30606(55.76) | 24284(44.24) | 0.23 | 20894(50.03) | 20867(49.97) | 0.00 |
| Black | 4272(66.43) | 2159(33.57) | 0.70 | 2107(49.63) | 2138(50.37) | 0.01 |
| Others | 3598(63.96) | 2027(36.04) | 0.58 | 1979(50.05) | 1975(49.95) | 0.00 |
| Location |  |  |  |  |  |  |
| Left | 25300(62.30) | 15307(37.70) | 0.51 | 14725(50.68) | 14328(49.32) | 0.03 |
| Right | 13176(50.02) | 13163(49.98) | 0.00 | 10255(49.05) | 10652(50.95) | 0.04 |
| Histological type |  |  |  |  |  |  |
| Adenocarcinoma | 33972(57.73) | 24877(42.27) | 0.31 | 21956(50.33) | 21672(49.67) | 0.01 |
| Mucinous adenocarcinoma | 4232(55.28) | 3424(44.72) | 0.21 | 2866(47.63) | 3151(52.37) | 0.09 |
| Signet ring cell cancer | 272(61.68) | 169(38.32) | 0.48 | 158(50.16) | 157(49.84) | 0.01 |
| Differentiated grade |  |  |  |  |  |  |
| Well | 3308(56.75) | 2521(43.25) | 0.27 | 2252(48.91) | 2352(51.09) | 0.04 |
| Moderate | 28762(57.74) | 21047(42.26) | 0.31 | 18657(50.81) | 18062(49.19) | 0.03 |
| Poor | 6406(56.65) | 4902(43.35) | 0.27 | 4071(47.13) | 4566(52.87) | 0.11 |
| T-classification ^a^ |  |  |  |  |  |  |
| T1 | 2753(58.38) | 1963(41.62) | 0.34 | 1851(49.69) | 1874(50.31) | 0.01 |
| T2 | 6956(56.16) | 5431(43.84) | 0.25 | 4854(49.35) | 4982(50.65) | 0.03 |
| T3 | 23900(57.71) | 17513(42.29) | 0.31 | 15267(50.59) | 14913(49.41) | 0.02 |
| T4 | 4867(57.73) | 3563(42.27) | 0.31 | 3008(48.37) | 3211(51.63) | 0.07 |
| N-classification ^a^ |  |  |  |  |  |  |
| N0 | 28016(55.8) | 22188(44.20) | 0.23 | 19212(50.39) | 18915(49.61) | 0.02 |
| N1 | 6711(61.16) | 4262(38.84) | 0.46 | 3903(48.57) | 4132(51.43) | 0.06 |
| N2 | 3749(64.99) | 2020(35.01) | 0.63 | 1865(49.10) | 1933(50.90) | 0.04 |
| nLN |  |  |  |  |  |  |
| 0 | 1862(58.76) | 1307(41.24) | 0.36 | 1197(49.83) | 1205(50.17) | 0.01 |
| 1-2 | 2087(54.21) | 1763(45.79) | 0.17 | 1515(46.77) | 1724(53.23) | 0.13 |
| 3-5 | 5280(54.56) | 4397(45.44) | 0.18 | 3649(47.69) | 4002(52.31) | 0.09 |
| 6-11 | 12476(55.44) | 10028(44.56) | 0.22 | 8581(51.19) | 8183(48.81) | 0.05 |
| ≥12 | 16771(60.44) | 10975(39.56) | 0.43 | 10038(50.43) | 9866(49.57) | 0.02 |
| Stage ^a^ |  |  |  |  |  |  |
| I | 8553(55.65) | 6815(44.35) | 0.23 | 6125(49.35) | 6287(50.65) | 0.03 |
| II | 19463(55.87) | 15373(44.13) | 0.24 | 13087(50.89) | 12628(49.11) | 0.04 |
| III | 10460(62.48) | 6282(37.52) | 0.52 | 5768(48.75) | 6065(51.25) | 0.05 |
| CT |  |  |  |  |  |  |
| No | 22518(50.17) | 22368(49.83) | 0.01 | 18575(49.58) | 18892(50.42) | 0.02 |
| Yes | 15958(72.34) | 6102(27.66) | 1.00 | 6405(51.27) | 6088(48.73) | 0.05 |
| RT |  |  |  |  |  |  |
| No | 31034(54.55) | 25860(45.45) | 0.18 | 22202(49.81) | 22375(50.19) | 0.01 |
| Yes | 7442(74.04) | 2610(25.96) | 1.10 | 2778(51.61) | 2605(48.39) | 0.06 |

^a^ Stage TNM, T, N-classification to 7^th^ edition of AJCC staging system.

All statistical tests were two-sided.

Abbreviations: CT: PSM: propensity score matching; SD: standardized difference; nLN: number of lymph nodes; CT: chemotherapy treatment; RT: radiotherapy treatment.

Left includes rectum, rectosigmoid junction, sigmoid colon, descending colon and splenic flexure.

Right includes transverse colon, hepatic flexure, ascending colon, cecum, and appendix.
