# Supplementary material for: Impact of old age on resectable colorectal cancer outcomes
Source: PeerJ. 2019 Feb 15;7:e6350. doi: 10.7717/peerj.6350 (PMC6378948; doi:10.7717/peerj.6350)
Supplement: Supplemental Information 5 [file peerj-07-6350-s005.docx]

| Risk Factors | Univariate analysis | | Multivariate analysis | |
| --- | --- | --- | --- | --- |
|  | HR (95%CI) | *P* ^b^ | HR (95%CI) | *P* ^b^ |
| Age |  |  |  |  |
| <70 | 1 |  | 1 |  |
| ≥70 | 1.37(1.33-1.42) | <0.001 | 1.41(1.36-1.45) | <0.001 |
| Gender |  |  |  |  |
| Female | 1 |  | 1 | - |
| Male | 1.16(1.13-1.20) | <0.001 | 1.26(1.22-1.30) | <0.001 |
| Marital status |  |  |  |  |
| Married | 1 |  | 1 | - |
| Unmarried | 1.25(1.18-1.32) | <0.001 | 1.22(1.15-1.28) | <0.001 |
| Divorced | 1.29(1.25-1.34) | <0.001 | 1.23(1.19-1.28) | <0.001 |
| Race |  |  |  |  |
| White | 1 |  |  |  |
| Black | 1.39(1.32-1.46) | <0.001 | 1.28(1.21-1.35) | <0.001 |
| Other | 0.86(0.81-0.92) | <0.001 | 0.87(0.82-0.92) | 0.012 |
| Location |  |  |  |  |
| Left | 1 |  | 1 |  |
| Right | 0.83(0.80-0.86) | <0.001 | 0.84(0.81-0.87) | <0.001 |
| Histology |  |  |  |  |
| [Adenocarcinoma](D:/360Downloads/Youdao/Dict/6.3.69.8341/resultui/frame/javascript:void(0);) | 1 |  | 1 |  |
| [Mucinous](D:/360Downloads/Youdao/Dict/6.3.69.8341/resultui/frame/javascript:void(0);) [adenocarcinoma](D:/360Downloads/Youdao/Dict/6.3.69.8341/resultui/frame/javascript:void(0);) | 1.07(1.02-1.12) | 0.007 | 1.03(0.98-1.08) | <0.001 |
| [Signet ring](D:/360Downloads/Youdao/Dict/6.3.69.8341/resultui/frame/javascript:void(0);) [cell](D:/360Downloads/Youdao/Dict/6.3.69.8341/resultui/frame/javascript:void(0);) [carcinoma](D:/360Downloads/Youdao/Dict/6.3.69.8341/resultui/frame/javascript:void(0);) | 2.39(2.05-2.79) | <0.001 | 1.35(1.16-1.58) | <0.001 |
| Differentiated grade |  |  |  |  |
| Grade I | 1 |  | 1 |  |
| Grade II | 1.24(1.16-1.31) | <0.001 | 1.05(0.99-1.12) | 0.104 |
| Grade III | 1.85(1.73-1.98) | <0.001 | 1.29(1.21-1.38) | <0.001 |
| T-classification ^a^ |  |  |  |  |
| T1 | 1 |  | 1 |  |
| T2 | 1.58(1.43-1.75) | <0.001 | 1.71(1.54-1.90) | <0.001 |
| T3 | 3.44(3.13-3.78) | <0.001 | 3.24(2.94-3.57) | <0.001 |
| T4 | 6.46(5.86-7.13) | <0.001 | 5.91(5.34-6.54) | <0.001 |
| N- classification ^a^ |  |  |  |  |
| N0 | 1 |  | 1 |  |
| N1 | 2.30(2.22-2.39) | <0.001 | 2.08(2.00-2.17) | <0.001 |
| N2 | 4.53(4.33-4.72) | <0.001 | 4.05(3.86-4.24) | <0.001 |
| nLN |  |  |  |  |
| 0 | 1 |  | 1 |  |
| 0-2 | 0.89(0.81-0.97) | 0.008 | 0.71(0.65-0.78) | <0.001 |
| 3-5 | 0.87(0.80-0.94) | <0.001 | 0.60(0.56-0.65) | <0.001 |
| 6-11 | 0.81(0.76-0.87) | <0.001 | 0.48(0.45-0.52) | <0.001 |
| ≥12 | 0.71(0.66-0.76) | <0.001 | 0.39(0.37-0.42) | <0.001 |
| CT |  |  |  |  |
| Yes | 1 |  | 1 |  |
| No | 1.80(1.74-1.86) | <0.001 | 0.96(0.93-1.00) | 0.064 |
| RT |  |  |  |  |
| Yes | 1 |  | 1 |  |
| No | 1.82(1.75-1.90) | <0.001 | 1.30(1.24-1.37) | <0.001 |

^a^ T classification according to 7^th^ AJCC staging system.

^b^ *P* values obtained from the χ2 test. All statistical tests were two-sided.

Abbreviations: PSM: propensity score matching; nLN: number of lymph nodes; CT: chemotherapy treatment; RT: radiotherapy treatment; HR: hazard ratio.

Left includes rectum, rectosigmoid junction, sigmoid colon, descending colon and splenic flexure.

Right includes transverse colon, hepatic flexure, ascending colon, cecum, and appendix.
