# Supplementary figures and images for: Impact of old age on resectable colorectal cancer outcomes
Source: PeerJ. 2019 Feb 15;7:e6350. doi: 10.7717/peerj.6350 (PMC6378948; doi:10.7717/peerj.6350)

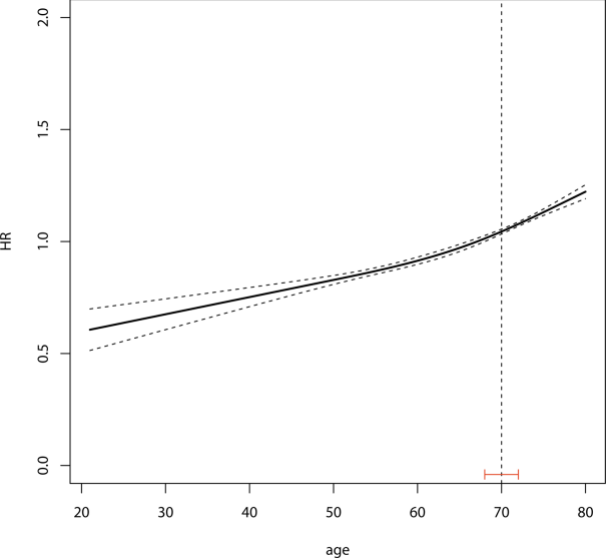

Supplement: Supplemental Information 6 [file peerj-07-6350-s006.pdf]

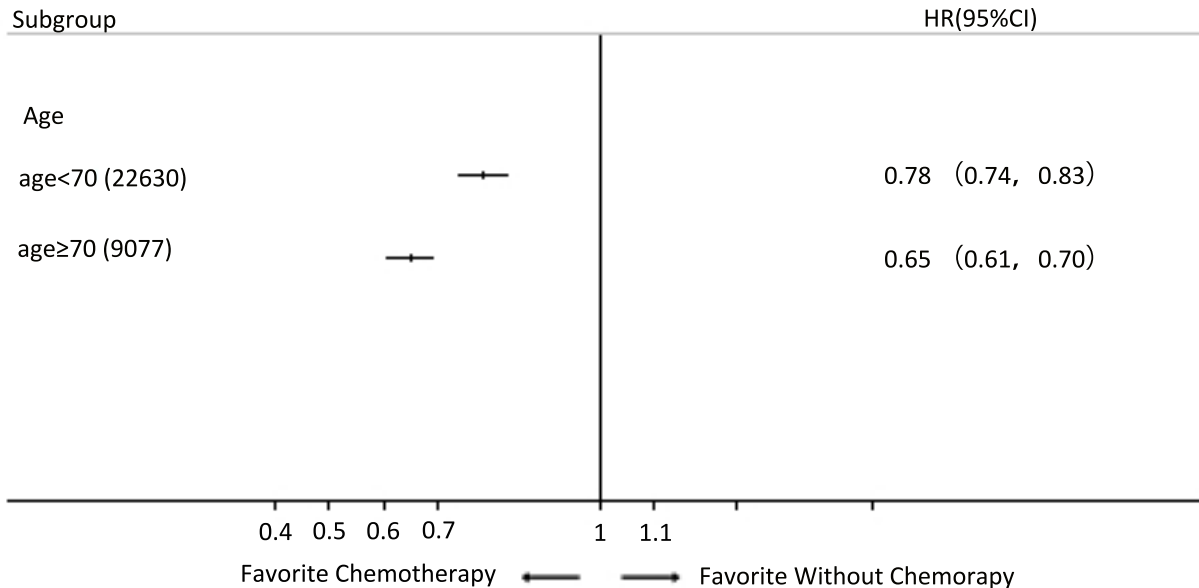

Supplement: Supplemental Information 7 [file peerj-07-6350-s007.pdf]

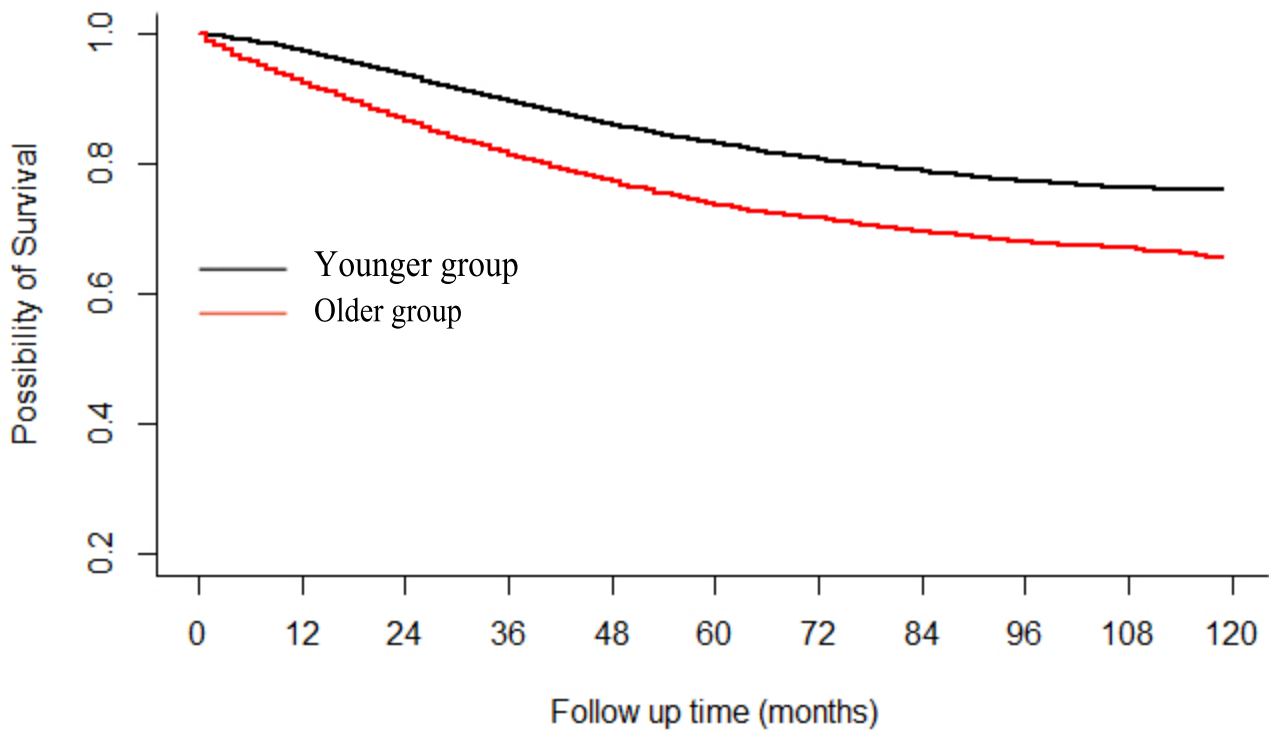

Supplement: Supplemental Information 8 [file peerj-07-6350-s008.pdf]

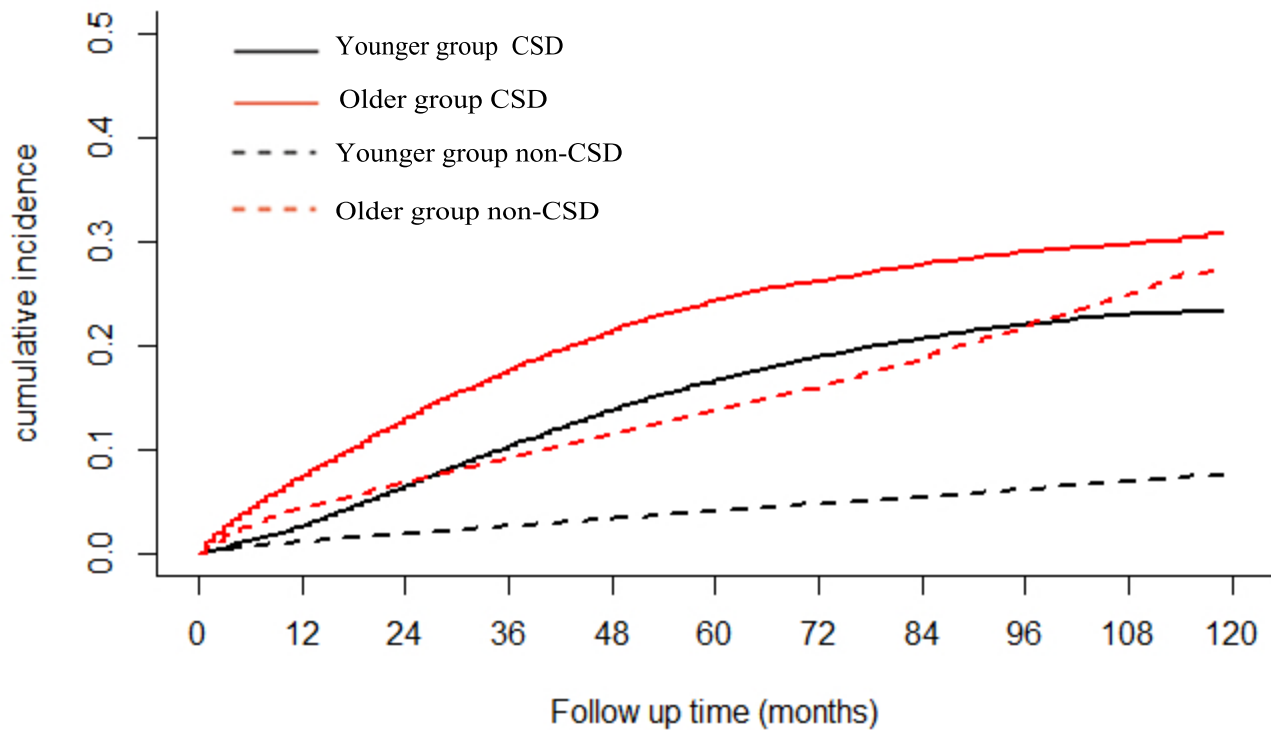

Supplement: Supplemental Information 9 — CSD: cancer-specific death. non-CSD: non-cancer-specific death. [file peerj-07-6350-s009.pdf]
