# Supplementary material for: Impact of old age on resectable colorectal cancer outcomes
Source: PeerJ. 2019 Feb 15;7:e6350. doi: 10.7717/peerj.6350 (PMC6378948; doi:10.7717/peerj.6350)
Supplement: Supplemental Information 10 [file peerj-07-6350-s010.pdf]

Inclusion and exclusion criteria in SEER (2004-2011)

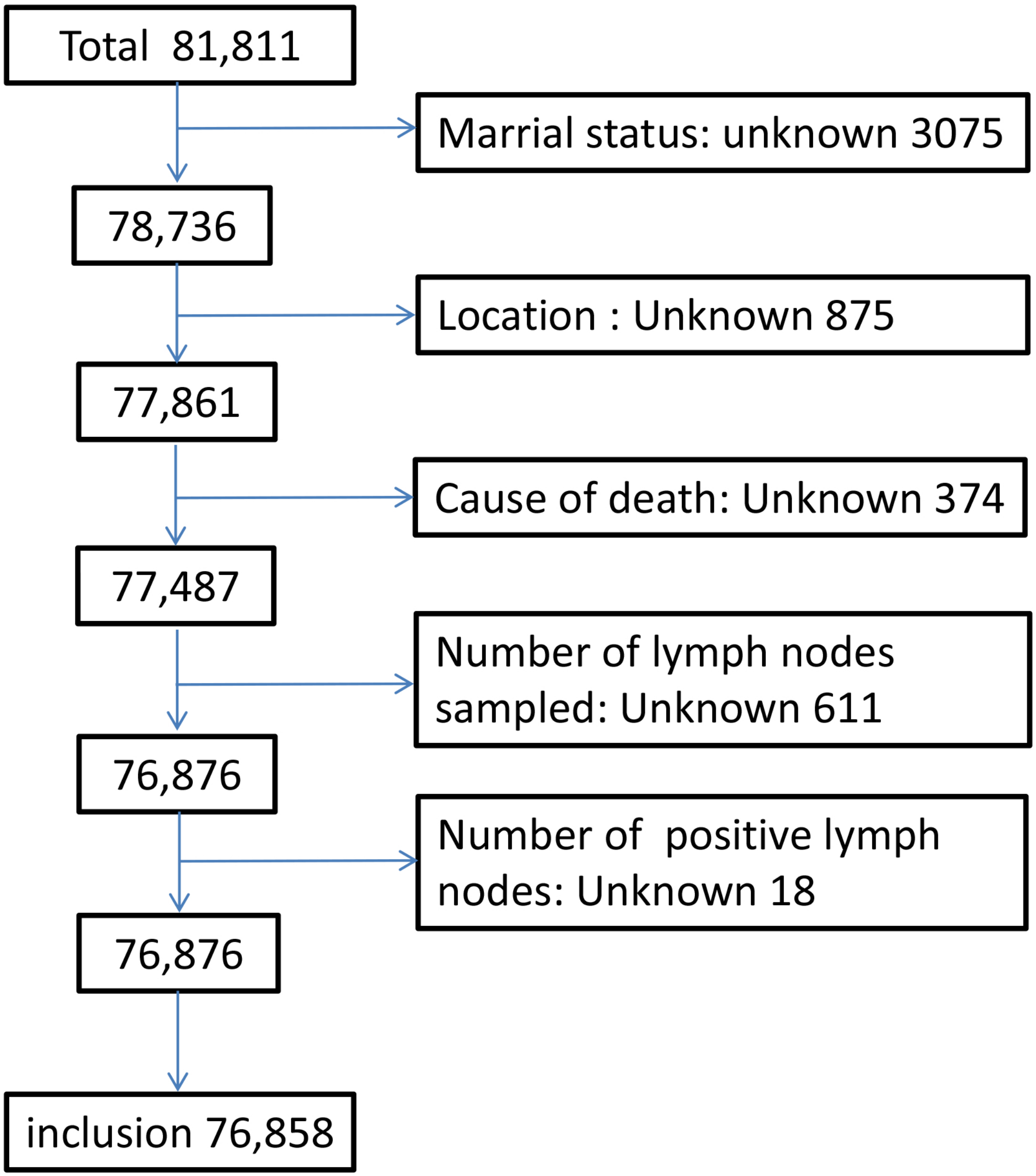

Selection in SEER

Site recode= Colon and Rectum
Age at diagnosis=20-80
Race=white, black and others
Histology=ICD-O-3 8140, 8480, 8481,84904
Year of diagnosis =2004-2011
Surgery code = 30-80
Survival months ≥1 months
Primary tumor number = 1
Classification M=M0
Classification N=N0-2
Classification T=T1-4
Grade=well, moderately, poorly
